# The Proton-Boron Reaction Increases the Radiobiological Effectiveness of Clinical Low- and High-Energy Proton Beams: Novel Experimental Evidence and Perspectives

**DOI:** 10.3389/fonc.2021.682647

**Published:** 2021-06-28

**Authors:** Pavel Bláha, Chiara Feoli, Stefano Agosteo, Marco Calvaruso, Francesco Paolo Cammarata, Roberto Catalano, Mario Ciocca, Giuseppe Antonio Pablo Cirrone, Valeria Conte, Giacomo Cuttone, Angelica Facoetti, Giusi Irma Forte, Lorenzo Giuffrida, Giuseppe Magro, Daniele Margarone, Luigi Minafra, Giada Petringa, Gaia Pucci, Valerio Ricciardi, Enrico Rosa, Giorgio Russo, Lorenzo Manti

**Affiliations:** ^1^ Istituto Nazionale di Fisica Nucleare (INFN), Sezione di Napoli, Naples, Italy; ^2^ Energy Department, Politecnico di Milano, and INFN, Sezione di Milano, Milan, Italy; ^3^ Istituto di Bioimmagini e Fisiologia Molecolare-Consiglio Nazionale delle Ricerche (IBFM-CNR), Cefalù, Italy; ^4^ Laboratori Nazionali del Sud (LNS), INFN, Catania, Italy; ^5^ Medical Physics Unit & Research Department, Centro Nazionale di Adroterapia Oncologica (CNAO) & INFN, Sezione di Pavia, Pavia, Italy; ^6^ Laboratori Nazionali di Legnaro (LNL), INFN, Legnaro, Italy; ^7^ Extreme Light Infrastructure (ELI)-Beamlines Center, Institute of Physics (FZU), Czech Academy of Sciences, Prague, Czechia; ^8^ Department of Biological, Chemical and Pharmaceutical Sciences and Technologies (STeBiCeF), Università di Palermo, Palermo, Italy; ^9^ Department of Mathematics & Physics, Università L. Vanvitelli, Caserta, Italy; ^10^ Radiation Biophysics Laboratory, Department of Physics “E. Pancini”, Università di Napoli Federico II, Naples, Italy; ^11^ The Sicilian Center of Nuclear Physics and the Structure of Matter (CSFNSM), Catania, Italy

**Keywords:** protontherapy, chromosome aberrations, proton-boron (B) fusion-enhanced proton therapy (PBFEPT), BSH, alpha-particle, cancer cell killing

## Abstract

Protontherapy is a rapidly expanding radiotherapy modality where accelerated proton beams are used to precisely deliver the dose to the tumor target but is generally considered ineffective against radioresistant tumors. Proton-Boron Capture Therapy (PBCT) is a novel approach aimed at enhancing proton biological effectiveness. PBCT exploits a nuclear fusion reaction between low-energy protons and ^11^B atoms, i.e. p+^11^B→ 3α (p-B), which is supposed to produce highly-DNA damaging α-particles exclusively across the tumor-conformed Spread-Out Bragg Peak (SOBP), without harming healthy tissues in the beam entrance channel. To confirm previous work on PBCT, here we report new in-vitro data obtained at the 62-MeV ocular melanoma-dedicated proton beamline of the INFN-Laboratori Nazionali del Sud (LNS), Catania, Italy. For the first time, we also tested PBCT at the 250-MeV proton beamline used for deep-seated cancers at the Centro Nazionale di Adroterapia Oncologica (CNAO), Pavia, Italy. We used Sodium Mercaptododecaborate (BSH) as ^11^B carrier, DU145 prostate cancer cells to assess cell killing and non-cancer epithelial breast MCF-10A cells for quantifying chromosome aberrations (CAs) by FISH painting and DNA repair pathway protein expression by western blotting. Cells were exposed at various depths along the two clinical SOBPs. Compared to exposure in the absence of boron, proton irradiation in the presence of BSH significantly reduced DU145 clonogenic survival and increased both frequency and complexity of CAs in MCF-10A cells at the mid- and distal SOBP positions, but not at the beam entrance. BSH-mediated enhancement of DNA damage response was also found at mid-SOBP. These results corroborate PBCT as a strategy to render protontherapy amenable towards radiotherapy-resilient tumor. If coupled with emerging proton FLASH radiotherapy modalities, PBCT could thus widen the protontherapy therapeutic index.

## Introduction

Protontherapy (PT) is a radiotherapy (RT) modality exploiting the favorable physical properties of accelerated charged particles (1). These deposit a low dose at the beam entrance, releasing most of their energy near the end of their range, the Bragg peak, which can be conformed to the tumor (Spread-Out Bragg Peak, SOBP). Hence, compared to conventional cancer radiotherapy (CRT) using high-energy photon/electron beams, PT reduces the integral dose to healthy tissues, which implies an overall lower risk of RT-induced secondary cancers, and grants greater precision in contouring the dose to the tumor (2, 3). On the other hand, the relative biological effectiveness (RBE) at tumor cell killing of clinical protons is similar to that of CRT (4), hence PT offers no obvious advantage against cancer radioresistance, a major cause of treatment failure (5). Conversely, carbon-ion based RT is more effective because of the higher linear energy transfer (LET) these particles exhibit in the SOBP (6), resulting in mostly clustered and poorly reparable DNA damage (7). However, radiobiological and cost-effectiveness issues still affect this particle-based RT approach (8).

Recently, Proton-Boron Capture Therapy (PBCT) has been proposed as a possible strategy to potentiate proton biological effectiveness (9). Conceptually similar to the long-known Boron-Neutron Capture Therapy (BNCT), where highly-DNA damaging high-LET particles are released by thermal neutrons interacting with ^10^B (10), PBCT exploits the nuclear fusion reaction p+^11^B→ 3α (p-B). The maximum probability for the p-B reaction occurs for low-energy protons, i.e. at an energy of around 675 keV (11), such as those slowing down across the tumor-confined SOBP in PT. Being emitted with an energy of around 4 MeV (12), which corresponds to a range of less than 30 μm and an initial LET of around 100 keV/μm in water, these α-particles can cause a highly localized pattern of clustered DNA damage in the tumor (13). At the same time, the high proton energies at the beam entrance prevent α-particles from being generated in healthy tissues. We obtained a first experimental demonstration of PBCT-assisted enhancement of proton biological effectiveness (14) using the ocular melanoma-dedicated 62-MeV clinical proton beam at INFN-Laboratori Nazionali del Sud in Catania (Italy). Sodium Mercaptododecaborate (BSH) was used as a boron carrier, at a nominal ^11^B concentration of 80 ppm. Prostate cancer DU145 cells were used to measure clonogenic survival along the SOBP and non-cancer mammary epithelial MCF-10A cells to measure chromosome aberration (CA) frequency at mid-SOBP.

Here we report novel work carried out at INFN-LNS on the yield and degree of complexity of CAs in MCF-10A cells exposed at the entrance and distal SOBP positions: complex CAs are a well-known biomarker of exposure to high-LET radiation (15, 16). Expression of proteins involved in DNA repair pathways was also studied in MCF-10A cells irradiated at mid-SOBP. Moreover, for the first time we used the high-energy clinical proton beam available at the Centro Nazionale di Adroterapia Oncologica (CNAO) in Pavia, Italy. CAs were revealed by FISH techniques (17): together with whole chromosome painting (WCP) of chromosomes 1 and 2, karyotype reconstruction and analysis by multicolor(m)-FISH were carried out to better evaluate the yield of complex chromosomal rearrangements (18). Proton biological effectiveness is increased in the presence of BSH at both clinical facilities. PBCT could therefore represent a clinically exploitable strategy to expand the range of tumors treatable by PT. Furthermore, if coupled with the emerging proton FLASH-RT regimes, seemingly associated with a reduction in normal tissue late-occurring adverse effects (19, 20), PBCT could contribute to further widening the PT therapeutic index.

## Materials and Methods

### Cell Lines

Details on the cell lines used in this study can be found in Cirrone et al. (14). Briefly, human prostate cancer DU145 cells were grown in RPMI medium supplemented with 10% fetal bovine serum, 1% of l-glutamine and 1% of penicillin/streptomycin. Human mammary epithelial MCF-10A cells required two DMEM/F12-based media as described by Debnath et al. (21): one for optimal growth, containing 5% horse serum, EGF (20 ng/ml), hydrocortisone (0.5 μg/ml), insulin (10 μg/ml) and cholera toxin (100 ng/ml); the other was used for routine subcultivation, devoid of all supplements but serum-enriched (20%) to quench the action of trypsin during cell resuspension and counting dilutions. Penicillin/streptomycin was added to both media (1%). Both cell lines were grown in standard tissue culture flasks kept in a humidified atmosphere of 5% CO_2_ in air at 37°C.

### Boron Carrier

As a boron carrier, sodium mercaptododecaborate (BSH) Na_2_B_12_H_12_S (purchased from Katchem Ltd. Czech Rep., CAS 144885-51-8), with naturally occurring boron isotopic abundance (80% ^11^B and 20% ^10^B), was used. Prior to irradiation, it was weighed out and thoroughly dissolved in the appropriate volume of cell growth culture medium. The final working concentration was 80 ppm of ^11^B by weight, which corresponds to approximately 0.17 mg/ml of BSH. To ensure sterility, BSH-containing medium was syringe-filtered (0.22-μm pores) before being added to cell cultures. The pre-treatment of cell cultures with BSH-enriched medium started about 6-8 hours before irradiation. BSH-treated cells were irradiated in the presence of boron, hence immediately before exposure, flasks were completely filled with the appropriate medium containing 0.17 mg/ml BSH. This was necessary in order to minimize cellular stress since flasks were irradiated in the vertical position at INFN-LNS or CNAO due to the horizontal incidence of the proton beams. The same procedure was followed for control flasks filled up with BSH-free medium. After irradiation, media were removed, and cells assayed as below specified.

### Irradiations

#### Clinical Low-Energy Proton Beamline

**Figure 1:**
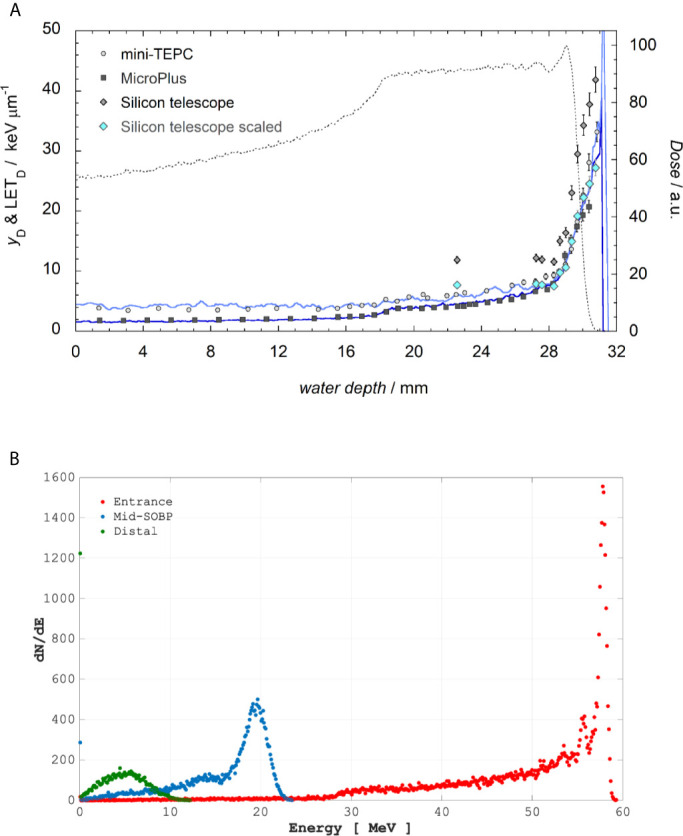
Top panel **(A)**: The LET-dose primary computed for only primary protons (blue line) and LET-dose total calculated considering also the contribution of generated secondary particles (indigo line) are reported. The dotted line represents the dose values measured in a water phantom with the Markus Chamber (mod. 3002). Experimental y_D_ values are obtained with the mini-TEPC (gray circles), MicroPlus (black squares), Silicon telescope (gray diamonds). Light blue diamonds represent the silicon data multiplied by a factor k=0.65. Bottom Panel **(B)**: Incident proton energy distribution corresponding to the positions where cells were placed as calculated by Hadrontherapy Geant4 advanced example.

Irradiations with the ocular melanoma-dedicated 62 MeV proton beamline were performed at the Centro di AdroTerapia e Applicazioni Nucleari Avanzate (CATANA) at INFN-LNS (Istituto Nazionale di Fisica Nucleare-Laboratori Nazionali del Sud) in Catania, Italy (22, 23). Details on cellular irradiation set-up can be found elsewhere (14). Briefly, a clinical SOBP with a modulation width of 11 mm and a penetration range of 29.5 mm in water was used. Cells were irradiated in three positions, i.e. entrance, mid- and distal SOBP, corresponding to water equivalent depths of 1, 23.76, and 29.45 mm, respectively, to which primary LET-dose values of 1.58, 5.02, and 16.32 keV/μm were associated. **Figure 1** shows the energy distribution within the SOBP at such positions and the LET at different positions along the SOBP, which was calculated by means of Monte Carlo simulations and microdosimetric measurements. The CATANA beamline was entirely simulated using the *Hadrontherapy* Geant4 advanced example (24, 25). The averaged LET-dose total and LET-dose primary were then calculated according to the procedure reported in (26). Microdosimetric spectra were measured with three detectors: mini-TEPC (27), Silicon telescope (28) and MicroPlus probe (29), and the dose mean lineal energy *y*_D_ was derived as the ratio between the mean energy imparted and the mean track length of primary protons in the irradiated sensitive volumes. The comparison of experimental *y*_D_ with the simulated LET is reported in **Figure 1A**. The best agreement between the averaged LET-dose total and the dose mean lineal energy *y*_D_ was found for the mini-TEPC, as was expected mainly because: i) the mini-TEPC is tissue-equivalent; ii) it has a smaller sensitive volume (1 μm water equivalent) than that simulated by the Silicon telescope, that is 3.3 μm, and by the MicroPlus probe, that is 17.2 μm; iii) it has a higher sensitivity (30, 31). The dosimetry of the clinical proton beam was performed just before each cellular irradiation with an uncertainty in absolute dose measurement within 1.5% as detailed elsewhere (14). Beam was extracted in air and cell culture tissue flasks were placed in front of the beam collimator on a remotely controlled in-house built sample holder (see **Supplementary Figure 1**). MCF-10A cells for CA studies were irradiated at the entrance and distal SOBP positions, with doses of 0.5, 2 and 4 Gy; in these cells, protein expression was studied after irradiation with 2 Gy at mid-SOBP.

#### Clinical High-Energy Proton Beamline

**Figure 2:**
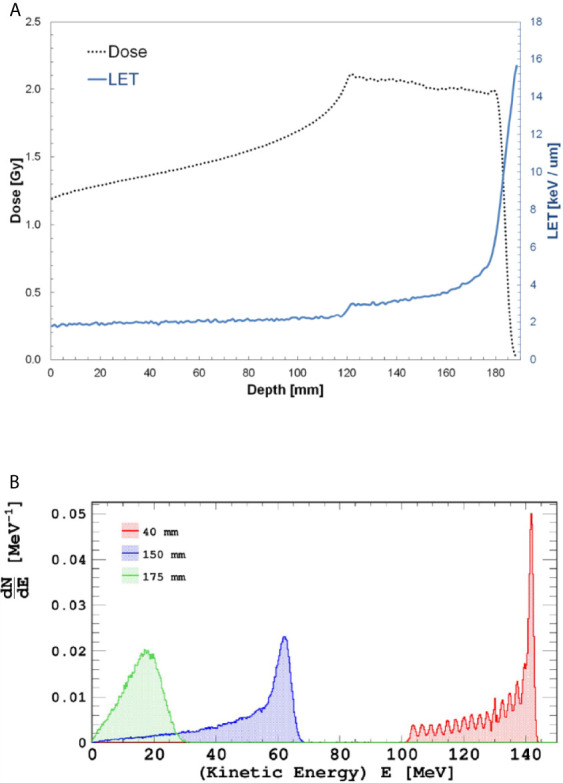
Top panel **(A)**: Dose- and LET-depth profiles for the CNAO SOBP used during the cellular irradiations. Bottom panel **(B)**: Incident proton distributions at the three positions where cells were irradiated; from right to left, energy distribution at entrance, mid-and distal SOBP.

Cell flask irradiations were performed in a 3-D motorized water phantom (MP3-P, PTW Freiburg, Germany) at three different water-equivalent depths (40, 150 and 175 mm), corresponding to the entrance plateau, middle and distal portion of a homogeneous SOBP, respectively. Dose-averaged LET values calculated using Monte Carlo FLUKA code (32) at the three reported depths were 1.96, 3.33 and 4.75 keV/μm, respectively as shown in **Figure 2A**. By analogy with the values reported in **Figure 1B** for the INFN-LNS SOBP, in **Figure 2B** the energy distributions of the incident proton beams within the SOBP are shown for the cell irradiation positions. A 60-mm width SOBP (120-180 mm in water) was achieved using 16 discrete proton energies (range: 131.5-164.8 MeV) generated by the CNAO synchrotron (33). Pencil beam scanning irradiation modality was adopted, similarly to the standard clinical scenario at CNAO (3-mm scanning step for proton beam spot). The absorbed dose to water was measured using a calibrated Farmer-type ionization chamber, following the IAEA TRS-398 code of practice (32, 34). The estimated relative standard uncertainty in the determination of absorbed dose to water under reference conditions was around 2% (34). Cell tissue culture flasks were placed in a water tank as shown in **Supplementary Figure 2**. DU145 cells were irradiated for measurement of radiation-induced cell death with doses of up to 4 Gy. To evaluate DNA damage complexity by analysis of radiation-induced CAs, MCF-10A cells were exposed to the same doses as at LNS-INFN, that is 0.5, 2 and 4 Gy.

### Measurement of Cellular Radioresponse

#### Clonogenic Assay

Cell death was measured in prostate cancer DU145 cells by loss of colony-forming ability. At least three replicates were used for each dose point and treatment condition (irradiation in the presence or the absence of BSH). Three independent experiments were carried out for each depth along the SOBP at CNAO. After incubation at 37°C in a 5% CO_2_ atmosphere for 12 days to allow for colony formation, cells were fixed and stained by 0.5% crystal violet dye in 85% methanol in water for 45 minutes at room temperature. Manually counted colonies with more than 50 cells were considered as survivors. Surviving fractions (SF) are obtained by dividing the number of colonies by the number of cells seeded at a given dose D, normalized by the plating efficiency (PE). Dose-response curves were thus constructed fitting the SF values to the linear-quadratic equation SF(D) = exp (–αD–βD^2^) by least square minimization according to modified Marquardt-Levenberg Algorithm for weighted nonlinear regressions (SigmaPlot v.14.0 SYSTAT). The fitting procedure was repeated setting α as the only free parameter if β was found consistent with zero.

#### Chromosome Aberration (CA) Assays

CAs were studied in MCF-10A cells at 36 h post irradiation by chemical induction of premature chromosome condensation (PCC). PCC was obtained following 30-min incubation in calyculin A (50 ng/ml, Sigma Aldrich) and collected by standard cytogenetic protocol as elsewhere described (35, 36), slightly modified for adherent cells. Detection of structural CAs was carried out by Fluorescence-in Situ Hybridization (FISH) techniques: Whole Chromosome Painting (WCP) and multicolor (m)-FISH (14, 37). For WCP, two pairs of homologous chromosomes were labelled with probes (MetaSystems, Germany) directed to chromosomes 1 and 2 emitting in the green (chromosome #1, XCP-1 FITC-conjugated probe) or red (chromosome # 2, XCP-2 orange) spectrum under UV light. Denaturation (72°C for 3 min) followed by hybridization (37°C for 4 h) of 72-h room-temperature aged slides was performed using the programmable HyBrite chamber system (Vysis, USA). After post-hybridization washes, chromosomes were counterstained by DAPI/antifade (250 ng/ml). For mFISH, the 24XCyte probe cocktail, made up of five fluorochromes by MetaSystems (CyTM5, DEAC, FITC, Spectrum OrangeTM, Texas Red), was applied to PCC spreads harvested as described above. A detailed protocol can be found in Cirrone et al. (14).

##### Aberration Scoring

Coded slides were viewed at an epi-fluorescence microscope (Axioplan2 imaging MOT, Carl Zeiss) connected to an automated platform (Metafer 4, MetaSystems) for slide scanning and color image acquisition. In the case of labelling by WCP, CAs were analyzed in FISH-stained chromosomes 1 and 2 on computer-stored images. All slides were blind-scored by the same scorer. All types of structural aberrations were scored separately and categorized in simple exchanges (i.e. translocations and dicentrics), either visibly structurally complete or incomplete, acentric excess fragments and complex exchanges, these being assessed as the result of an exchange involving not less than three breaks in at least two chromosomes (37, 38). For the study’s purpose, we considered the frequency of all chromosome exchanges, calculated as the ratio between all exchange-type aberrations (simple plus complex, both reciprocal and non-reciprocal) and the number of cells scored; frequencies for complex-type CAs were also reported separately. No centromere probe was used but centromeres were clearly distinguishable as bright bands under DAPI illumination. Not less than 500 chromosome spreads were counted for each dose, SOBP position and boron treatment status, with more than 1,000 PCC being analyzed for unirradiated controls. Frequency of aberration exchanges was fitted to the equation Y = Y_0_+αD+βD^2^. For mFISH analysis, karyotype reconstruction was manually carried out on PCC spreads acquired and processed using the system described above and by means of the ISIS imaging software (MetaSystems, Germany), which attributes a false color pattern depending on overlap signals intensity, according to 24XCyte labeling scheme provided by the manufacturer. Not less than 100 karyotypes were analyzed for each experimental point. As in the case of WCP, all types of aberrations were scored separately and categorized as simple exchanges (either complete or incomplete) and complex exchanges. To classify the degree of complexity in the chromosomal rearrangements due to high-LET α-particles, the number of chromosomes and the number of breaks involved per complex exchange were evaluated, similar to Lee et al. (39), and presented as frequencies (ratios to the number of cells scored). A Poisson statistics was assumed to evaluate standard errors (SE) on aberration mean frequencies and significance between frequency data was assessed by Z-test using the StatCalc 3.02 software (Acastat Software, USA).

#### Western Blotting

Total cell lysates from BSH-treated and untreated MCF-10A cells were obtained by using a solubilization and denaturation buffer (8 M Urea, 4% CHAPS, 65 mM DTE, 40 mM Tris) supplemented with protease and phosphatase inhibitors (Sigma-Aldrich). Protein concentration was determined by the Bradford protein assay (Bio-Rad). Aliquots of 30 µg cell lysates were subjected to the SDS polyacrylamide gel electrophoresis (SDS-PAGE), performed in a range of gel concentrations from 6 to 12% according to the molecular weight of the proteins to be separated. Protein transfer was carried out into nitrocellulose membrane (HyBond ECL, Amersham) by electroblotting at 100 V for 60 min at 4°C in the transfer buffer (25 mM Tris, 190 mM Glycine, 20% Methanol). Membranes were treated with a blocking solution (5% non-fat dehydrated milk in 0.05% TBST) for 1 h at room temperature and then incubated with a primary antibody in the appropriate dilution in a 0.05% TBST solution with 1% dry milk, overnight with stirring at 4°C. The primary antibodies used were the following: DNA Polymerase beta (Novus Biologicals), Phospho-ATR (Abcam), Phospho-XPA (Thermo Fisher Scientific), Ku70/XRCC6 (Novus Biologicals), Phospho-γH2AX BioLegend), β-Actin (Sigma-Aldrich). Following incubation with the appropriate secondary antibody peroxidase-linked, chemiluminescent reactions were detected by using the Chemidoc system as per manufacturer’s instructions (Biorad). Protein quantification was performed with the ImageMaster 2D Platinum software (Amersham Biosciences) by densitometric analysis of the immune-reactive bands. The expression of β-actin was used as an internal standard for data normalization, the signal of each protein band was normalized to the densitometric value of β-actin and the protein quantification expressed as fold-change in respect of the control sample (untreated).

## Results

### Irradiations at the Low-Energy INFN-LNS Facility

In previous experiments, the non-tumorigenic MCF-10A cell line was used to assess enhancement of DNA damage by BSH in the form of CAs in samples exposed at the mid-SOBP position of the 62-MeV proton beam of LNS-INFN (14). Here, for the first time, MCF-10A cells were irradiated at the beam entrance and at the distal SOBP position as detailed in 2.3.1 and CA yield and complexity were analyzed. The purpose was to investigate the clinically-relevant dependence on proton energy, hence on depth along the SOBP, of boron-mediated radiosensitization due to the p-B reaction. The expression of DNA damage-activated repair proteins was also studied at mid-SOBP.

#### Chromosome Aberration (CA) Induction and Complexity Along the INFN-LNS Proton SOBP

**Figure 3:**
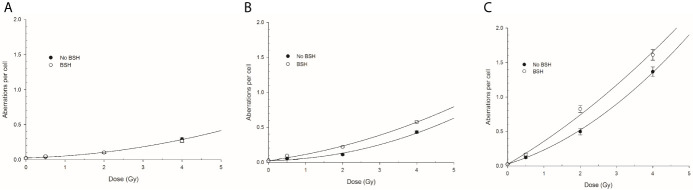
Chromosome aberration frequency measured by WCP analysis in cells exposed along the INFN-LNS proton beam SOBP in the presence or the absence of BSH: left panel **(A)** refers to the entrance position, central panel **(B)** to mid-SOBP data from Cirrone et al. (14), and right panel **(C)** to distal position. Error bars depict SE of at least three independent replicates. Data were fitted to a linear-quadratic function Y=Y_0_+αD+βD^2^ with Y_0_ being the CA frequency in unirradiated cells.

**Figure 4:**
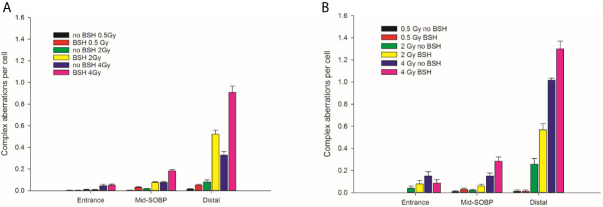
Frequency of complex-type aberrations as revealed by WCP, left panel **(A)**, or by mFISH, right panel **(B)**, as a function of dose and position along the INFN-LNS proton beam SOBP for samples irradiated in the presence or absence of BSH. Data from previous experiments (14) obtained for mid-SOBP are also presented for comparison. Error bars depict SE of the mean from at least three independent replicates.

CA frequencies were measured by both WCP and mFISH analysis. **Figure 3** shows the frequency of all CA types revealed by WCP as a function of proton dose from cells exposed at the entrance and distal SOBP positions. For sake of comparison, data previously obtained from cells exposed at mid-SOBP are also shown (**Figure 3B**). A dose-dependent increase in the amount of CAs in non-BSH treated cells can be observed at all positions. After 4 Gy of protons, a 4.5-fold and 3-fold elevation in the frequency of aberrations per cell was recorded at the distal position (**Figure 3C**) in comparison to entrance (**Figure 3A**) and mid-SOBP (**Figure 3B**) positions, respectively. More importantly, DNA damage is increased by the p-B reaction. Proton irradiation results in a significant elevation of CA frequency in BSH-treated cells compared to their counterparts irradiated in the absence of BSH at the distal position (**Figure 3C**), while no effect due to the boron carrier is observed at the beam entrance (**Figure 3A**). Specifically, at the distal position, for BSH-treated samples, about 0.83 and 1.61 aberrations per cell are recorded after 2 Gy and 4 Gy of protons compared to 0.50 and 1.37 found in non-BSH samples at the same doses, with a BSH-associated fold change of 1.7 and 1.2, respectively (**Figure 3C**). A greater proportion of complex-type rearrangements as detected by WCP and mFISH was measured in PCC spreads at the distal SOBP compared to entrance and mid-SOBP (**Figure 4**), reflecting the increase in LET of the primary beam (**Figure 1A**). Furthermore, the absolute values of such complex-type CAs were greatest in BSH-treated irradiated cells and when mFISH technique was used. No such a difference could be measured when scoring was carried out with either WCP or mFISH on PCC spreads from cells irradiated at the beam entrance. At the latter position, in fact, very low frequencies of complex exchanges and no BSH dependence were found, even at the highest dose used, with 4 Gy yielding less than 0.05 complex CAs per cell after WCP analysis (**Figure 4A**). Conversely, at the distal position, the measured frequency of complex-type CAs as revealed by WCP in BSH-treated cells was greater than that measured for their non-BSH counterparts at all proton irradiation doses, such an increase being more than 3-fold already at a dose as low as 0.5 Gy (0.052 in BSH-treated samples vs. 0.014 in non BSH-treated ones); after 2 Gy and 4 Gy, about 0.52 and 0.91 complex CAs per cell were observed following proton irradiation in the presence of the boron compound compared to around 0.08 and 0.33 scored in non-BSH samples (**Figure 4A**). BSH per se did not influence the overall yield of CAs in unirradiated MCF-10A cells, with a baseline frequency similar to that previously reported (14).

**Figure 5:**
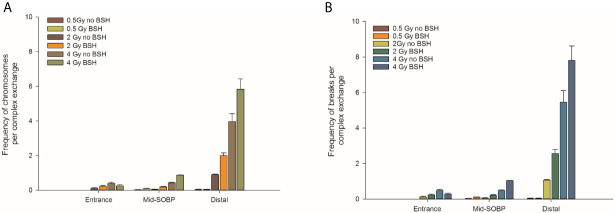
Classification of complex exchanges revealed by mFISH analysis in terms of number of chromosomes involved in complex exchanges per cell, left panel **(A)**, and number of breaks in complex exchanges per cell, right panel **(B)** for MCF-10A cells irradiated at entrance, mid, and distal positions of the INFN-LNS low-energy clinical proton beam.

By allowing detection of chromosome exchanges involving all chromosomes, mFISH-based karyotyping is best suited to accurately quantify LET-dependent aberration complexity. In fact, a greater amount of complex-type CAs than that revealed by WCP was observed when this technique was used (**Figure 4B**) in all irradiated samples, particularly at the distal SOBP position. Moreover, mFISH data confirmed a greater occurrence of complex-type CAs in BSH-treated samples compared to those exposed to the proton beam in the absence of the boron carrier at mid- and distal SOBP positions, with no significant difference due to BSH at the beam entrance. In particular, at the distal SOBP the frequency of complex CAs after 2 Gy and 4 Gy proton irradiation reached 0.6 and 1.3 aberrations per cell in PCC spreads from BSH-treated cells compared to frequency values of 0.26 and 1.0 measured in non-BSH samples, respectively (**Figure 4B**). To further characterize the degree of complexity in the aberrations scored by mFISH, the number of chromosomes involved in complex exchanges per cell and the number of breaks in complex exchanges per cell were also measured (**Figure 5**) for the newly acquired data at entrance and distal; previously acquired data obtained at mid-SOBP (14) were also re-assessed in light of such parameters. Complex exchanges scored in BSH-treated irradiated cells at mid- and distal SOBP positions consistently show a higher frequency of chromosomes per complex exchange (**Figure 5A**) and of breaks per complex exchange (**Figure 5B**) than that found in complex exchanges detected in cells exposed to protons in the absence of the boron carrier. For example, following 4 Gy proton irradiation at distal, almost 6 chromosomes per complex exchange per cell and 8 breaks per complex exchange per cell were found in BSH-treated cells compared to figures of 4 chromosomes and 5 breaks measured in complex CAs found in non-BSH samples. No appreciable difference could be instead observed for samples irradiated at entrance for either parameter. Overall, these results are consistent with a boron-mediated increase in the yield and complexity of proton irradiation-induced DNA damage as a result of the p-B reaction.

#### Western Blotting Analysis of DNA Damage Repair Machinery

**Figure 6:**
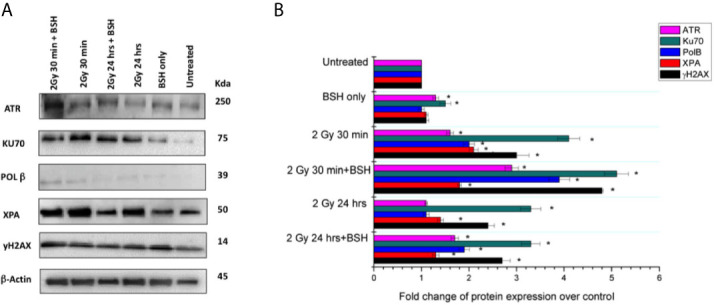
Western Blotting analysis of DNA damage response in the MCF-10A cell line irradiated with 2 Gy of proton beam with or without BSH, studied at two time points: 30 min and 24 h post-irradiation. **(A)** Western Blot gel example with the studied proteins; **(B)** Fold change of protein expression. The data shown are representative of three independent experiments and are expressed as the mean ± standard error of the mean (SEM). The significance level compared to the untreated sample was set to p < 0.05 and displayed with the asterisk (*).

In order to detect the expression of proteins involved in DNA damage repair and to highlight putative differences due to the presence of BSH, Western Blotting (WB) analyses were performed in MCF-10A cells after irradiation with 2 Gy of protons at mid-SOBP. Two time points of analysis were chosen to examine the activation and downregulation of the DNA Damage Response (DDR), which usually reaches a peak of activity at 30 min and gradually declines over the course of 24 hours (40), thus samples were assayed at 30 min and 24 h past the exposure (**Figure 6A**). Protein quantification was performed by densitometric analysis using the β-actin expression as housekeeping protein for data normalization: expression values for each protein are reported as fold change with respect to controls (**Figure 6B**), as described in the method section. ATR (Ataxia Telangiectasia Mutated and Rad-3) is generally activated when both single- and double-strand breaks occur (41). WB analysis of ATR at 30 min after irradiation showed an increase of protein expression with a fold change of 1.6 in the 2 Gy sample and 2.9 in the 2 Gy + BSH sample, while at 24 h a fold change of 1.1 and 1.7, respectively, was observed. For Ku70, a DNA-binding protein involved in the non-homologous end joining pathway (NHEJ) as reviewed in (42), WB analysis showed an increase in expression by a factor of 4.1 and 5.1 in the 2 Gy and 2 Gy + BSH samples 30 min post irradiation, respectively. However, no difference between the two samples was observed at 24 h, although a 3.3-fold increased expression of Ku70 was measured. Polymerase Beta (POLB) plays a key role in Base Excision Repair (BER), which is activated in response to base lesions and abasic sites that occur during single-strand repair (43). An increased POLB expression of 2.0 and 3.9 at 30 min post-irradiation was revealed in the 2 Gy and 2 Gy + BSH samples, while in the samples assayed at 24 h an increase by a factor of 1.1 and 1.9, respectively, was observed. Similar to POLB, XPA is activated by single-strand breaks and in particular during Nucleotide Excision Repair (NER) (44). The XPA WB analysis revealed an increased expression by 2.1 and 1.8 in the 2 Gy and 2 Gy+BSH samples at 30 min after irradiation, respectively, and an increase of 1.4 and 1.3 at 24 h. We also investigated the expression of the phosphorylated form of the histone H2AX. At 30 min post-irradiation, an increase of 3.0 and 4.8 in the 2Gy and 2 Gy+BSH samples, respectively, was observed, while at 24 h the protein expression increased by a factor of 2.4 and 2.7, respectively. Altogether, these results suggest a BSH-mediated increase in the DNA damage response machinery. However, additional analysis will be performed in the near future to further clarify the role of BSH in inducing a higher DNA damage yield with respect to proton irradiation alone.

### Irradiations at the High-Energy CNAO Facility

For the first time, the pre-clinical feasibility of the PBCT approach was tested at the synchrotron-generated proton SOBP routinely used to treat deep-seated tumors at CNAO. Loss of colony-forming ability and aberration induction were investigated to assess whether proton irradiation in the presence of BSH, similarly to what was found at the lower-energy LNS-INFN PT facility, resulted in an increase of cancer cell death due to complex DNA damage caused by the high-LET α-particles from the p-B reaction. To this end, prostate cancer DU145 cells and non-tumorigenic MCF-10A cells were irradiated at three different positions along the CNAO SOBP as specified in 2.3.2.

#### Clonogenic Dose-Response Curves

**Figure 7:**
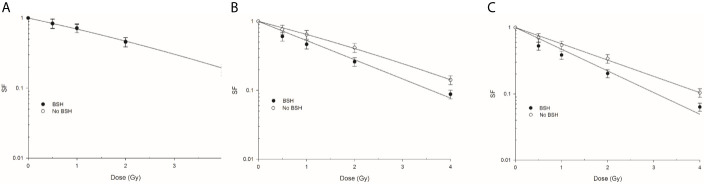
Clonogenic survival of prostate cancer DU145 cells irradiated along the CNAO proton beam SOBP. Effect of the presence or absence of 80 ppm of ^11^B from BSH on the survival fraction (SF) in three positions: Left panel **(A)** entrance, **(B)** mid SOBP, and right panel **(C)** distal. Error bars depict SE of at least three independent replicates. Data were fitted to a linear-quadratic function SF=exp-(αD+βD^2^).

**Figure 7** shows the clonogenic dose-response curves obtained from DU145 cells exposed at beam entrance, mid- and distal SOBP positions in the presence or absence of the boron carrier BSH. As shown by the curve fitting parameters (**Table 1**), the effectiveness at cell killing generally increases with depth along the SOBP, this being maximal at the distal position where the clonogenic dose-response curve of non-BSH cells is best fitted by a pure exponential function. This is in line with the increase in LET as shown in **Figure 2A**. More interestingly, at the entrance position of the SOBP (**Figure 7A**) no difference in surviving fraction (SF) was observed between BSH-treated and non-BSH samples. At mid- and distal SOBP positions, instead, SF values are significantly lower for cells irradiated in the presence of the boron carrier than those recorded for cells irradiated in the absence of BSH (**Figures 7B, C**), with fitting curves from BSH-treated cells at such positions exhibiting a pure exponential decrease with dose (**Table 1**). An SF_2_ value of about 0.42 was found for non-BSH samples compared to a value of 0.26 as measured in BSH-treated cells at mid-SOBP (**Figure 7B**). Boron treatment did not affect clonogenic survival of unirradiated cells as PE values did not differ between BSH-treated and untreated cells and were on average around 55% (data not shown). To quantify the BSH-induced increase in proton irradiation-induced cell killing, the Dose-Modifying Factor at 10% level (DMF_10_) was calculated and was about 1.3 at mid-SOBP (**Table 1**): the presence of BSH thus resulted in an increase of the effectiveness by proton radiation dose to reduce the SF of DU145 cells to 0.1 by about 30% compared to pristine proton irradiation. A slight but not statistically significant increase was observed for DMF_10_ at distal position. These results are consistent with the p-B reaction as being responsible for an increase of the effectiveness of the CNAO clinical proton beamline at tumor cell killing.

**Table 1:** Linear-quadratic fitting parameters and DMF_10_ for survival curves obtained after irradiation of DU145 along the CNAO proton beam SOBP.

Fitting parameters	α (Gy^-1^)	β (Gy^-2^)	DMF_10_
Entrance	0.346 ± 0.017	0.017 ± 0.005	
Mid-SOBP No BSH	0.421 ± 0.034	0.017 ± 0.010	–
Mid-SOBP BSH	0.640 ± 0.037	–	1.29 ± 0.14
Distal No BSH	0.565 ± 0.012	–	–
Distal BSH	0.752 ± 0.064	–	1.33 ± 0.12

#### Chromosome Aberration Induction and Complexity Along the CNAO Proton SOBP

**Figure 8:**
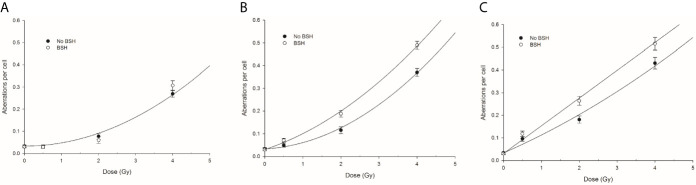
CA frequency measured by WCP analysis in cells exposed along the CNAO proton beam SOBP: left panel **(A)** refers to entrance position, central panel **(B)** to mid-SOBP, and right panel **(C)** to distal SOBP. Error bars depict SE of at least three independent replicates. Data were fitted to a linear-quadratic function Y=Y_0_+αD+βD^2^.

Proton irradiation-induced CAs were scored in PCC spreads from MCF-10A cells exposed at the beam entrance, mid- and distal SOBP position of the CNAO clinical beamline in the presence or the absence of the boron carrier BSH. The dose-response curves for total aberration frequencies show that the yield of DNA damage generally increases with dose and, at each dose, with depth in BSH-untreated cells, the presence of boron significantly exacerbating proton irradiation-induced DNA damage at mid- and distal SOBP positions but not at the beam entrance (**Figure 8**). In fact, after 2 Gy, between 0.08 and 0.06 aberrations per cell were measured in BSH-treated and untreated cells, respectively, at the beam entrance (**Figure 8A**); at such a dose, the recorded CA frequency was 0.12 and 0.18 at mid-SOBP and distal in PCC from cells irradiated in the absence of boron, while rising to 0.18 and 0.26 when irradiation had occurred in the presence of BSH (**Figures 8B, C**). At the highest dose used, i.e. 4 Gy, about 0.30 aberrations per cell were measured at the entrance, irrespective of boron presence, while rising to 0.37 and 0.43 at mid-SOBP and distal, respectively, for cells irradiated in the absence of BSH. At the same dose and positions, BSH-treated cells exhibited 0.49 and 0.52 aberrations per cell (**Figure 8**). As seen for CA measured following irradiation at INFN-LNS, BSH did not exert any cytotoxic action per se as similar baseline frequencies were observed (data not shown), in keeping with values previously reported (14).

**Figure 9:**
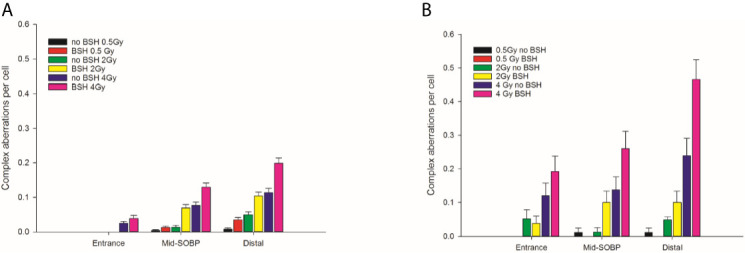
Frequency of complex CA as revealed by WCP, left panel **(A)**, or mFISH, right panel **(B)**, as a function of dose and position along the CNAO proton beam SOBP for samples irradiated in the presence or absence of BSH.

**Figure 10:**
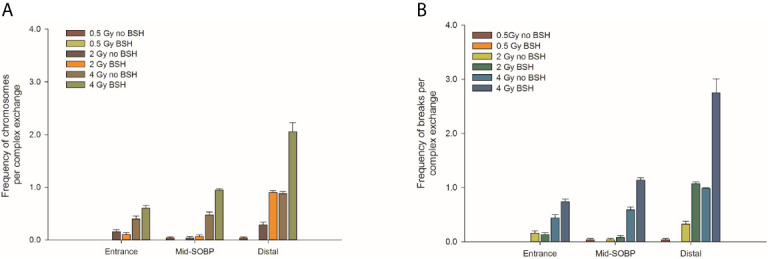
Classification of complex exchanges revealed by mFISH analysis in terms of frequencies of number of chromosomes, left panel **(A)**, and number of breaks [right panel **(B)**] involved; for MCF-10A cells irradiated at entrance, mid, and distal positions of the CNAO clinical proton beam.

The yield of complex chromosomal rearrangements as well the degree of complexity associated with such exchanges were measured along the SOBP (**Figures 9** and **10**). In particular, the frequency of complex-type aberrations was determined by WCP (**Figure 9A**) and mFISH (**Figure 9B**) techniques. At mid-SOBP, following 2 Gy and 4 Gy of proton irradiation, WCP-based analysis showed 0.07 and 0.13 complex CAs per cell in BSH-treated samples compared to values of 0.01 and 0.08 in their non-BSH treated counterparts at the same doses (**Figure 9A**). These values rose to 0.10 and 0.20 following 2 Gy and 4 Gy at distal SOBP in BSH-treated cells compared to complex CA frequencies of 0.05 and 0.11 detected in PCC from non-BSH samples. At the entrance position, no complex aberrations could be found by WCP following either 0.5 Gy or 2 Gy, while similar values were measured after 4 Gy between BSH and non-BSH samples (**Figure 9A**). Analysis by mFISH confirmed the occurrence of a greater proportion of complex rearrangements in BSH-treated samples compared to PCC from cells that had been irradiated in the absence of BSH, with overall higher absolute frequency values in all scored samples due to the karyotype-wide sensitivity of the technique (**Figure 9B**). Indeed, mFISH analysis made possible to ascertain that a greater degree of complexity was associated with the greater occurrence of complex exchanges found in samples irradiated in the presence of BSH, showing a higher number of chromosomes involved per complex exchange per cell (**Figure 10A**) and a higher number of breaks per complex exchange per cell (**Figure 10B**) compared to non-BSH samples: after 4 Gy, for example, twice as many chromosomes per complex exchange could be found in BSH-treated samples compared to non-BSH samples at mid- and distal SOBP (**Figure 10A**). The frequency of breaks per complex exchange was also twice as much after 4 Gy at mid-SOBP in BSH-treated samples compared to non-BSH ones, becoming 3-fold greater at distal SOBP following the same dose (**Figure 10B**).

**Figure 11:**
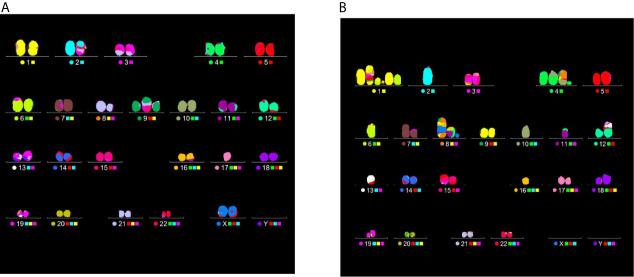
Examples of CA analysis by mFISH. Images depict karyotypes from samples irradiated at the CNAO beamline with 4 Gy of protons in the presence of BSH and show typical aberrations types found at two positions, i.e. a simple exchange between chromosomes 2 and 17 from a cell exposed at the entrance [left panel, **(A)**] and several complex rearrangement detected in a PCC spread from a cell irradiated at the distal position [right panel, **(B)**]. See main text for details.

**Figure 11** clearly demonstrates the different degree of aberration complexity revealed by mFISH between samples scored from cells exposed to the same dose, in this case 4 Gy, but at different positions of the CNAO beamline: in **Figure 11A** a translocation is visible in the karyotype obtained from a cell exposed at the entrance. **Figure 11B**, on the other hand, refers to a karyotype reconstructed from a cell irradiated at the distal position containing several aberrations, namely: a complex exchange between chromosomes 1, 6, and 9, entailing 5 breaks; a complex exchange between chromosomes 4, 8, and 10 (with 3 breaks); a complex exchange between chromosomes 8, 11, 16, and the X chromosome (for a total of 6 breaks); a dicentric between chromosomes 12 and 13. Excluding the latter, which is a simple-type exchange, the number of chromosomes and breaks involved in the complex exchanges for this particular karyotype amounts to 10 and 14, respectively (**Figure 11B**).

## Discussion

Hadrontherapy is an advanced cancer radiotherapy (RT) modality using accelerated charged particle beams. The charged particles’ inverted dose-depth profile (Bragg curve) translates in greater sparing of healthy tissues and improved precision in dose delivery thanks to the tumor-conformed Spread-Out Bragg Peak (SOBP) compared to conventional radiotherapy (CRT) based on high-energy photon/electron beams (1, 45). Currently, protons and carbon ions are being used. However, clinical protons exhibit a relative biological effectiveness (RBE) at tumor cell killing similar to that of CRT, with a fixed value of 1.1 universally adopted in treatment planning. Carbon ion beams, on the other hand, have a higher linear energy transfer (LET), around 50 keV/μm compared to the 4-5 keV/μm of protons at mid-SOBP (33), leading to mostly clustered DNA damage, whose poor reparability leads to a greater RBE compared to both PT and CRT. However, carbon-ion RT is affected by radiobiological uncertainties on long-term consequences from normal-tissue damage and the presence of a fragmentation tail leading to unwanted dose beyond the SOBP (8, 46). Hence, PT represents a safer choice to lower the overall risk of RT-induced secondary cancers, especially in the case of pediatric patients (3, 47). Moreover, carbon-ion clinical facilities are still considerably more expensive than PT ones (8). As a result, PT is rapidly growing worldwide (see statistics periodically updated by the Particle Therapy Co-operative Group or PTCOG, accessible online at www.ptcog.ch), despite ongoing debate as evidence-based medicine critics dispute its overall cost-effectiveness (48). However, because cancer radioresistance, either intrinsic or acquired during RT, is a major cause for treatment failure by favoring metastasization and disease recurrence, increasing proton biological effectiveness remains an attractive prospective in hadrontherapy. In fact, although PT is generally regarded as ineffective against radioresistant cancers, there exists evidence for a peculiarly different radiobiological behaviour exhibited by protons compared to photons (49), with reports showing greater radiosensitization ability than that expected based solely on LET, for instance in causing ROS-mediated damage to cancer stem cells (50). Together with the known higher RBE at the distal position of clinical proton SOBP (51), this has led to urge the implementation of a variable RBE in PT (52). Indeed, several biology-based radiosensitizing strategies are being explored, such as combining particle therapy with targeting of specific molecular pathways involved in radioresistance, as recently reviewed by Konings et al. (53), although more pre-clinical research is needed.

One alternative to potentiate proton biological efficacy is based on nuclear physics and stems from the proposed adoption of a binary approach, termed Proton-Boron Capture Therapy or PBCT (9), in which a highly localized emission of high-LET α-particles resulting from the interaction between low-energy protons and atoms of ^11^B (p-B reaction, in brief) is supposed to severely damage cancer cells’ DNA. We obtained a first pre-clinical demonstration of PBCT at the relatively low-energy clinical proton beamline of the INFN-LNS (Catania, Italy) reporting a significant reduction in the colony-forming ability of prostate cancer DU145 cells irradiated in the presence of the boron carrier BSH (14). Non-cancer human mammary epithelial MCF-10A cells were used to study DNA damage (in the form of chromosome aberrations, CAs) in order to avoid the confoundingly high baseline CA frequency from genomically unstable cancer cells: the presence of BSH resulted in an elevation of CA induction, and particularly of complex-type exchanges, compared to cells irradiated with protons alone at mid-SOBP (14). In this work, we present further *in vitro* results on the biological effects of the p-B reaction triggered by proton irradiation in clinical settings. Novel data on CA induction and complexity, as well as on repair protein expression, were obtained at the INFN-LNS facility. Moreover, for the first time, experiments were carried out at the high-energy proton beamline routinely used for deep-seated cancer protontherapy at the Centro Nazionale di Adroterapia Oncologica (CNAO), Pavia (Italy).

### The p-B Reaction Enhances the Yield and Complexity of Proton-Induced DNA Damage Along the SOBP of the INFN-LNS Proton Beamline

Structural chromosomal rearrangements reflect both the amount and the pattern of energy deposition events by ionizing radiation on the (sub)micrometric scale. Therefore, their frequency correlates with overall radiation-induced DNA damage, and an increased proportion of complex aberration types reflects exposure to higher radiation LET, such as that of the α-particles from the p-B reaction. CAs as revealed by WCP and mFISH were analyzed in MCF-10A cells irradiated at the beam entrance and distal position at the INFN-LNS therapeutic proton beamline. The yield of CAs was greater in BSH-treated samples compared to that measured in cells exposed to protons in the absence of the boron carrier at the distal position (**Figure 3C**) while no BSH-related difference was observed in cells irradiated at the beam entrance (**Figure 3A**). Moreover, the CA frequency measured in BSH-treated MCF-10A cells at the distal SOBP position was also greater for all radiation doses than that previously recorded at the mid-SOBP (14) and shown in **Figure 3B**. These results, therefore, confirm those showing a depth-dependent increase in BSH-mediated enhancement of clonogenic cell killing in DU145 cells at this facility (14) and further corroborate the notion that proton-induced DNA damage is exacerbated by the p-B reaction. Since the latter is triggered by low-energy protons, that is at around 700 keV, it can be expected that as protons slow down across the SOBP, the magnitude of the DNA-damaging effect brought about by the reaction-generated α-particles will increase with depth along the SOBP, i.e., with the decrease in the mean incident proton energy. As shown by the spectra reported in **Figure 1B**, at the distal position, the incident proton energy distribution is centered around about 5 MeV while being about 20 MeV at mid-SOBP and slightly less than 60 MeV at the beam entrance. It is worth noticing that also for non-BSH treated samples, the frequency of CAs increases with depth at all doses, such an increase being more marked at the distal position compared to either entrance or mid-SOBP, which reflects the significant differences between LET values at such depths, i.e. about 16 keV/μm vs. 1.6 and 5 keV/μm, respectively (**Figure 1A**). This is in keeping with a greater proton effectiveness at cell killing towards the distal part of the SOBP as found by Chaudhary et al. (51) at the same facility. Indeed, the sharp increase in RBE at the distal position used in this work may result in a dose-dependent “saturation effect”, partially masking the fold increase due to the p-B reaction: this can explain why the measured 6.5-fold increase in complex-type CA frequency measured by WCP labelling after 2 Gy was reduced to a factor of 2.7 after 4 Gy at the distal SOBP (**Figure 4A**). At this position, a similar attenuation in the enhancing effect on damage complexity of the p-B reaction is observed at the highest dose used following mFISH analysis (**Figure 4B**).

As mentioned, the rationale underlying PBCT as a means to increase proton biological effectiveness is the exploitation of the high-LET α-particles generated by the p-B reaction because the highly spatio-temporally clustered nature of the lesions created by such densely ionizing particles at the DNA level will compromise cellular repair proficiency. Moreover, compared to sparsely ionizing radiation, more chromosomal domains will be likely to be traversed by a single α-particle track, which will in turn cause multiple DNA breakage sites. This will manifest itself as an increase in the overall complexity of the chromosomal rearrangements arising from mis-repair of such damage (8). Thus, to gather further evidence in support of the radiosensitizing action of the p-B reaction, measurement of the yield of complex-type CAs was carried out by two FISH-based techniques, Whole Chromosome Painting (WCP) and multicolor(m)-FISH karyotyping. The latter, in particular, was used because it allows a detailed quantification of the number of chromosomes and breaks involved in each complex-type chromosomal rearrangement, thereby providing an accurate estimate of the degree of complexity. In fact, the higher LET at the distal position caused a significant increase in complex CAs in all irradiated samples in this work. However, both WCP and mFISH analysis concurred in showing that irradiation of MCF-10A cells at the distal position in the presence of BSH resulted in a much greater occurrence of complex CAs than in non-BSH treated samples, with mFISH being able to unveil a consistently greater proportion of such exchanges compared to those detected by WCP (**Figure 4**). No effect due to the presence of BSH could be instead measured at the beam entrance, where, as expected on the basis of the low LET of the primary proton beam, the proportion of complex exchanges never exceeded 0.15 aberrations per cell as measured at the highest dose used (4 Gy) by mFISH analysis. Conversely, at distal SOBP, 2 Gy of protons yielded a frequency of complex aberrations per cell in BSH-treated samples that is twice as much as that measured by the same technique at the same dose in non-BSH samples **(** **Figure 4B**). Furthermore, the proportion of complex CAs scored by WCP in BSH-treated compared to non-BSH samples increased with the depth along the SOBP, rising from a factor of around 4 at mid SOBP to more than 6 at distal after 2 Gy. More significantly, mFISH analysis allowed the detection at mid- and distal SOBP, but not at the beam entrance, of a greater number of chromosomes per complex exchange per cell in BSH-treated cells compared to non-BSH samples at all doses (**Figure 5A**). Accordingly, the frequency of breaks per complex exchange was found to be consistently higher in cells irradiated in the presence of the boron carrier (**Figure 5B**). The appropriateness of FISH techniques, and specifically of combinatorial painting (mFISH), to detect high LET radiation-associated chromosome damage complexity has been long supported (54, 55). Although a straightforward comparison with existing results obtained for *in vitro* cellular exposures to external beams of α-particles may hold little significance considering the binary process under investigation here, our results are consistent with the level of CA complexity expected following similar LET values for these particles (56) as well as other ions (39). Overall, these data allow to conclude that the yield of proton-induced DNA damage is significantly increased by the presence of the boron carrier BSH at therapeutically relevant positions along the SOBP, i.e. at the mid- and distal SOBP, but not at the beam entrance, where the healthy tissue would lie. Moreover, based on the CAB (Chromosome, Arm, Break) criterion for assessing chromosomal damage complexity (38), the results on the occurrence of complex CAs in irradiated BSH-treated cells point to the high-LET α-particles from the p-B reaction as the most likely underlying mechanism.

#### The p-B reaction Results in an Increased Upregulation of the DNA Damage Response (DDR) Machinery at the Mid-SOBP of the INFN-LNS Proton Beamline

The effect of the presence of the boron carrier BSH on the expression of key molecules belonging to specific DNA repair pathways was investigated by means of Western Blotting (WB) assays following the exposure of MCF-10A cells at the mid-SOBP at the INFN-LNS facility. In particular, we analyzed the expression of 5 proteins: The X-Ray Repair Cross Complementing 6 (XRCC6/KU70) involved in NHEJ, the Xeroderma Pigmentosum Group A-Complementing Protein (XPA) involved in NER, the Polymerase Beta (POLB) involved in BER, the Ataxia Telangiectasia and Rad3-Related kinase (ATR), involved in both SSBs and DSBs repair. In addition to this pool of DNA damage biomarkers, we also analyzed the expression of the phosphorylated form of histone H2AX (γH2AX), since it represents a well-known early marker of DNA DSBs (57). Since tumor cells often display defective or not functional DNA repair mechanisms, the non-tumorigenic MCF-10A cell line, commonly used as a healthy control epithelial cell line (58, 59) was thus used to study the activation of DDR pathways.

ATR is activated upon DSB formation and represents a master regulator of HR; moreover, it phosphorylates the histone γH2AX downstream of a DNA damage event (60, 61). Thus, ATR expression can be related to DSB levels in response to proton irradiation in combination with the p-B reaction. In our analysis, the expression of ATR increased at the time point of 30 min after irradiation (2 Gy) in the BSH-treated samples (**Figure 6**). This is consistent with the peak in the onset of activated γH2AX foci at this time (62). As expected, ATR signal decreased at 24 hours post irradiation in the non-BSH samples, still remaining higher in samples pretreated with BSH. One of the master regulators of NHEJ is the heterodimer formed by two proteins, Ku70/Ku80, thus the expression of Ku70 can be indicative of the triggering of non-homologous recombination. NHEJ, which is prevalent in mammalian cells, however, should not be considered as an exclusive mechanism of DSB repair and its activation can be simultaneous and also modulate the HR alternative pathway of DSB repair (63). As expected, Ku70 expression increased 30 min post irradiation in the presence of BSH, thus suggesting that NHEJ is likewise activated in response to DNA DSB during proton irradiation, like HR. Similar to the ATR modification, the Ku70 levels at 24 hours post irradiation remained high, with and without the BSH pre-treatment, respect to the controls. The elevated levels of DSB repair markers even at 24 hours are in keeping with the findings from CA analysis, due to the error prone DSB machinery, especially of the NHEJ.

On the other hand, ionizing radiation also induces DNA SSBs, BER being considered as one of the main pathways involved in the repair of SSB sites (64). One of the most important enzymes involved in BER is the Polymerase Beta which is required to remove the 5´-deoxyribose-5-phosphate of an abasic site and to fill the gap between DNA strands (65). As for the DSB repair pathways, also BER was affected by the presence of the boron carrier and POLB expression was higher in BSH-treated cells, meaning that both double- and single-strand break repair systems were active together at the same time and contributed to DDR. Since HR and NHEJ converge to the phosphorylation of the histone H2AX, also its phosphorylated form was increased after BSH treatment in our cell samples. Unexpectedly, NER, and in particular its master regulator XPA, did not show a level of expression correlated to BSH treatment, hence NER could be a less-activated mechanism of SSB repair following irradiation in the presence of BSH.

### The p-B Reaction Increases the Biological Effectiveness of the High-Energy CNAO Therapeutic Proton Beamline

The presence of 80 ppm of ^11^B from BSH during irradiation resulted in an increase in clonogenic cell death of prostate cancer DU145 cells (**Figure 7**) and in an increase in the yield and complexity of DNA damage assayed by FISH-labelled CAs (**Figures 8**–**10**) in non-tumorigenic MCF-10A epithelial cells along a clinical proton SOBP at CNAO. Such effects were observed at mid- and distal SOBP positions but not at the beam entrance.

#### The Presence of BSH Causes In Vitro Enhancement of Radiation-Induced Cancer Cell Death at the Clinical CNAO Proton Beamline

DU145 cells were exposed at three depths, corresponding to the beam entrance, mid- and distal positions, along a clinical 180-mm SOBP (**Figure 2**). Clonogenic dose-response curves show that the presence of BSH led to an enhancement of radiation-induced cell death at mid- and distal SOBP positions (**Figures 7B, C**). No BSH-dependent difference in measured surviving fraction (SF) was instead observed for samples irradiated at the beam entrance (**Figure 7A**). As shown by the curve fitting parameters for the non-BSH treated samples reported in **Table 1**, proton effectiveness moderately increases with depth along the SOBP, in accordance with the increase in radiation LET (**Figure 2A**), being greater at the clinically relevant mid- and distal positions. BSH-related radiosensitization slightly increased, albeit not significantly, from mid to distal position, while being null at beam entrance. Thus, SF_2_ values in BSH-treated cells were 0.26 and 0.20 at mid- and distal SOBP compared to 0.42 and 0.34 measured for non BSH-treated cells, respectively. The level of radiosensitization induced by BSH was quantified by the Dose-Modifying Factor at the 10% cell survival level (DMF_10_). This was around 1.3 at both mid- and distal positions (1.29 and 1.33, respectively, as shown in **Table 1**), indicating an increase of about 30% in dose-dependent proton biological effectiveness at cancer cell killing by the p-B reaction. DMF_10_ values from our previous experiments with the same cell line were 1.46 and 1.75 at the mid- and distal SOBP positions at the INFN-LNS facility beamline (14), which was also used in this work in relation to the DNA damage results reported above (The p-B Reaction Enhances the Yield and Complexity of Proton-induced DNA Damage Along the SOBP of the INFN-LNS Proton Beamline). The fact that the magnitude of the radiosensitizing effect due to the p-B reaction was slightly smaller at CNAO can be explained by the overall higher energy distributions of the incident proton beam along the SOBP compared to those at INFN-LNS: **Figures 1B** and **2B** clearly show that at mid-SOBP, for example, mean proton energy distributions were centered at around 60 MeV at CNAO and at around 20 MeV in the case of INFN-LNS. At the distal position, the differences between the beams from the two facilities in terms of LET (**Figures 1A** and **2A**) and mean incident energy (**Figures 1B** and **2B**) become even wider, thereby accounting for the more pronounced differences seen in terms of both overall radiosensitivity of non-BSH samples and the enhancing effects of the p-B reaction at this position between the two facilities. Thus, the greater LET exhibited at the distal position by the lower energy proton beamline at INFN-LNS leads to a steeper dose-response curve compared to that measured for samples exposed at the distal SOBP at CNAO, as shown by the value for the fitting alpha parameter of 0.314 ± 0.022 Gy^-1^ found at INFN-LNS (14) compared to the value of 0.565 ± 0.012 Gy^-1^ found in this study (**Table 1**); the differences in mean incident proton energy, on which triggering of the p-B reaction depends, are exemplified by the above-mentioned differences between the DMF_10_ values found at the distal position of the two beamlines.

#### Increase in Chromosome Damage Yield and Complexity in BSH-Treated MCF-10A Cells Irradiated at the High-Energy Clinical CNAO Proton Beamline

The presence of BSH exacerbated proton-induced DNA damage in MCF-10A cells irradiated along the CNAO proton SOBP. DNA damage was evaluated by measuring the frequency of CAs. The positions where MCF-10A cells were exposed were the same as those used for irradiation of the cancer DU145 cells assayed for cell death. At mid- and distal SOBP positions, but not at the beam entrance, a significant increase in the overall yield of FISH-painted CAs, scored in chemically induced PCC spreads, was measured following irradiation in the presence of BSH **(** **Figure 8**). The role of the p-B reaction is supported by the greater proportion of complex-type aberrations (**Figure 9**) as well as the higher degree of complexity (**Figure 10**) that accompanied these complex exchanges as found in BSH-treated cells at mid- and distal SOBP positions, which implicates exposure to high-LET radiation, such as the α-particles emitted by the nuclear fusion reaction between slowing down protons and the ^11^B atoms contained in BSH. No evidence of an increase in overall CA frequency nor of complex-type CAs was observed in MCF-10A cells irradiated at the highest proton energy, i.e. at the beam entrance. Compared to the results found following irradiation at the lower energy proton beam line, i.e., INFN-LNS, similar values for both the overall CA frequency and that of complex-type CAs were found for samples irradiated at the entrance and the mid-SOBP positions. A lower yield of all types of CAs, and particularly of complex ones, was instead observed following irradiation at the distal SOBP position of the CNAO beamline. This is in keeping with the lower LET associated with the latter, which is less than 5 keV/μm (**Figure 2A**), compared to an LET value of around 16 keV/μm for the distal SOBP at INFN-LNS (**Figure 1A**). Together with the data on clonogenic survival, the *in vitro* results on aberration yield and complexity obtained at CNAO are consistent with those from INFN-LNS and concur to support the potential usefulness of the binary PBCT strategy to enhance the effectiveness of a high-energy clinical proton beam.

### The Proton-Boron Capture Therapy (PBCT) Approach and Its Perspectives in Protontherapy

Marrying the advantageous ballistic properties presented by accelerated proton beams with a greater biological effectiveness by means of the PBCT approach is an arguably attractive perspective. This could make it possible, in principle, to achieve greater tumor local control as a consequence of dose-escalated/hypofractionated regimens in protontherapy (PT) treatment planning while mitigating the risk of adverse normal-tissue toxicity. More importantly, enhancing PT effectiveness could also expand the range of cancers eligible for treatment by PT by including those refractory to CRT. The first, and thus far to the best of the authors’ knowledge, only experimental proof by Cirrone et al. (14) that the p-B reaction can indeed increase the biological effectiveness of a clinical proton beam, has sparked interest on PBCT as demonstrated by recently published studies (66–70). It is worth pointing out that all these studies are in silico ones, speculating exclusively on the basis of theoretical calculations and modelling. Moreover, opposed to Ganjeh and Eslami-Kalantari (70), whose simulations using a phantom model of brain tumor agree with the potential benefits deriving from the p-B reaction, the recurrent criticism put forward by those arguing against the meaningfulness of PBCT is that the increase in the deposited dose within the target volume by the α-particles from the p-B reaction would be negligible, hence insufficient to elicit a measurable, clinically relevant effect (68, 69). However, it has been long known that macroscopically absorbed dose is just one factor on which the radiobiological efficiency of ionizing radiation depends; other physical parameters reflecting the inherently inhomogeneous pattern of energy deposition events at the micro- and nanometric scale, such as particle track structure, impact the fate of irradiated cells. Thus, DNA damage complexity, rather than the mere dose-dependent quantity of inflicted DNA damage, is mostly responsible for the increase in RBE observed with densely ionizing radiations (13, 18, 71). As far as low-energy α-particles are concerned, for example, exhaustive work compiled by Tracy et al. (72) substantiate how a single particle traversal through a cell’s nucleus is highly effective at cell killing, far beyond the actual dose being there deposited. Such effectiveness is mainly the consequence of the highly clustered DNA damage being generated along the track, which can be detected as complex chromosomal rearrangements (15, 17), in line with our results. Furthermore, even more difficult-to-model phenomena can influence cellular and tissue response to ionizing radiation. It has been known for over two decades that so-called non-targeted effects may play an important role in determining biological responses to ionizing radiation: these are not quantitatively reconcilable with the directly induced initial damage (e.g. radiation-induced genomic instability) nor confined to physically hit cells, as is the case for the wide range of radiation-induced bystander effects (RIBEs) recently reviewed by Kadhim and Hill (73). There exists indeed consensus that the magnitude of such non-targeted effects increases with increasing radiation LET (74, 75). High-LET exposure, such as that following α-particle irradiation, appears to be particularly prone to elicit RIBEs (75–77) mediated by signaling factor(s) being released by directly hit cells that can propagate for considerable distances from the site of the initial energy deposition event (78, 79). In fact, the impact of such non-targeted effects becomes especially relevant in low-fluence scenarios, when relatively low numbers of particles are involved, hence fewer cells are likely to be directly hit (80). This is, in principle, precisely the scenario corresponding to our experimental set up, where a relatively low fluence of α-particles is deemed to be generated by the p-B reaction. Indeed, the contribution of RIBEs as a concomitant mechanism assisting the enhancement of proton biological efficacy by PBCT is being currently investigated by us, together with the use of other ^11^B carriers, namely boronophenylalanine (BPA), in line with work from Hideghéty et al. (67), whose overall positive assessment on the potential of PBCT was accompanied by suggestions on the use of more clinically viable boron delivery agents based on a thorough assessment of the experience accumulated in BNCT. With regard to this, it is important to highlight that the choice of the agent (BSH) and concentration (80 ppm) used in this study as well as in the previous experimental work on PBCT (14) was indeed based on the BNCT experience with similar ^10^B-enriched compounds (81–85), the BSH molecule having a high boron content in its natural isotopic abundance (80% ^11^B, 20% ^10^B). In fact, being well aware of the poor penetrability of BSH into the cell, compared for example to the above-mentioned BPA, irradiations were always performed on cells that had been pre-treated with BSH and that were in BSH-containing medium at the moment of the irradiation.

The results presented here provide radiobiological evidence-based proof of the feasibility of the PBCT approach in clinical PT settings showing that the p-B reaction is able to exacerbate proton irradiation-induced cyto- and genotoxicity. It can therefore be speculated that not only could PBCT increase anti-tumor response by PT, but it may further widen its therapeutic ratio if coupled with the so-called FLASH-RT regimes that envisage dose rates far exceeding those used by conventional RT (e.g. above 40 Gy s^-1^). Wilson et al. (86) have recently reviewed the latest experimental evidence and the perspectives for a clinical translation of the reported benefits by FLASH-RT in terms of significantly reduced normal-tissue toxicity in face of identical tumor control rates. As expected, FLASH dose rates are being increasingly explored for therapeutic applications, both radiobiologically and technically, also with proton beams (19, 20, 87–89). Moreover, recent developments in the field of optically driven particle acceleration techniques have made the availability of extremely high-intensity laser sources a concrete possibility that could be exploited in the near future for ultra-high dose rate laser-driven PT (90, 91) such as at the ELIMAIA beamline, part of the ELI consortium (Prague, Czech Rep). In this context, the International Biophysics Collaboration for applied biomedical research has been recently launched with the aim of networking the growing number of particle accelerator facilities (92), either based on the above-mentioned laser-matter interaction or on conventional beam production and transport techniques that are being upgraded towards unprecedented beam intensities (e.g. FAIR at the GSI, Germany). This will provide an ideal platform for investigating what could represent a new frontier in evidence-based PT: achieving increased tumor control, even in radioresistant cancers currently untreated by PT, owing to the PBCT approach, and fewer late-arising normal tissue reactions through the FLASH dose delivery regimes.

### Conclusions

Using both low- and high-energy clinical proton beamlines, we demonstrated that Proton-Boron Capture Therapy increased proton biological efficacy. Our data point to the highly radiobiologically effective α-particles generated by the interaction of slowing down protons with ^11^B atoms exclusively across the SOBP-enveloped tumor volume as the main underlying, but not exclusive mechanism, as other peculiar biological responses elicited by such particles, may also play a role. A significant increase in clonogenic cell death, with a Dose-Modifying Factor at 10% cell survival of around 1.3, which was accompanied by an upregulation of the DNA damage response machinery and an increased yield of chromosomal aberrations, particularly of those highly complex in nature deriving from misrepaired clustered DNA lesions, were recorded in the samples irradiated in the presence of the boron agent at mid- and distal SOBP positions. No excess damage was measured at the beam entrance, in line with the predicted dependence on proton energy of the p-B reaction. PBCT might therefore be a therapeutically viable option to enhance PT biological effectiveness. These results, albeit encouraging, are far from being conclusive as data shown here need to be strengthened by additional *in vitro* preclinical data, using more clinically suitable boron delivery agents, and then carefully designed *in vivo* studies. Nevertheless, coupled with fast-growing FLASH-PT modalities, PBCT could re-shape protontherapy as currently administered making it even safer and more effective.

## Data Availability Statement

The raw data supporting the conclusions of this article will be made available by the authors, without undue reservation.

## Author Contributions

PB, CF, and LMa carried out cell survival and chromosome aberration measurements at INFN-LNS and CNAO and performed data analysis. SA, GAPC, VC, and GPe performed microdosimetry measurements at INFN-LNS. MCa, FPC, GIF, LMi, GPu, and GR carried out DNA damage measurement and data analysis at INFN-LNS and contributed the corresponding part in the original draft. RC, GAPC, and GPe supervised beam dosimetry at INFN-LNS. LG and DM provided overall conceptual contribution. MCi, GM, and AF assisted with dosimetry, irradiation and access to CNAO. GC acquired funding. VR and ER assisted with chromosome aberration analysis. LMa conceptualized the study and wrote the first draft of the manuscript. All authors contributed to the article and approved the submitted version.

## Funding

This work was financially supported by 2019-21 INFN grant NEPTUNE (Nuclear process-driven Enhancement of Proton Therapy, UNravEled) and MIUR PRIN2017 PBCT. This work was also supported by European Structural and Investment Fund and the Czech Ministry of Education, Youth and Sports (Project International mobility MSCA-IF IV FZU - CZ.02.2.69/0.0/0.0/20-079/0017754). This research was partially supported by the project “TP01010035 PHysics, Applied Research for Novel TECHnologies” funded by Technology Agency of the Czech Republic.

## Conflict of Interest

Author LG, DM and GAPC declare a potential conflict of interest being patent holder of the following invention: “DEVICE AND METHOD FOR IMAGING AND ENHANCED PROTONTHERAPY TREATMENT USING NUCLEAR REACTIONS. Application no.: EP16178280.0 – 1109/3266470.

The remaining authors declare that the work described in this paper was conducted in the absence of any specific relationship that could be construed as a potential conflict of interest.
