# Supplementary material for: The Proton-Boron Reaction Increases the Radiobiological Effectiveness of Clinical Low- and High-Energy Proton Beams: Novel Experimental Evidence and Perspectives
Source: Front Oncol. 2021 Jun 28;11:682647. doi: 10.3389/fonc.2021.682647 (PMC8274279; doi:10.3389/fonc.2021.682647)
Supplement: Supplementary file 1 [file DataSheet_1.pdf]

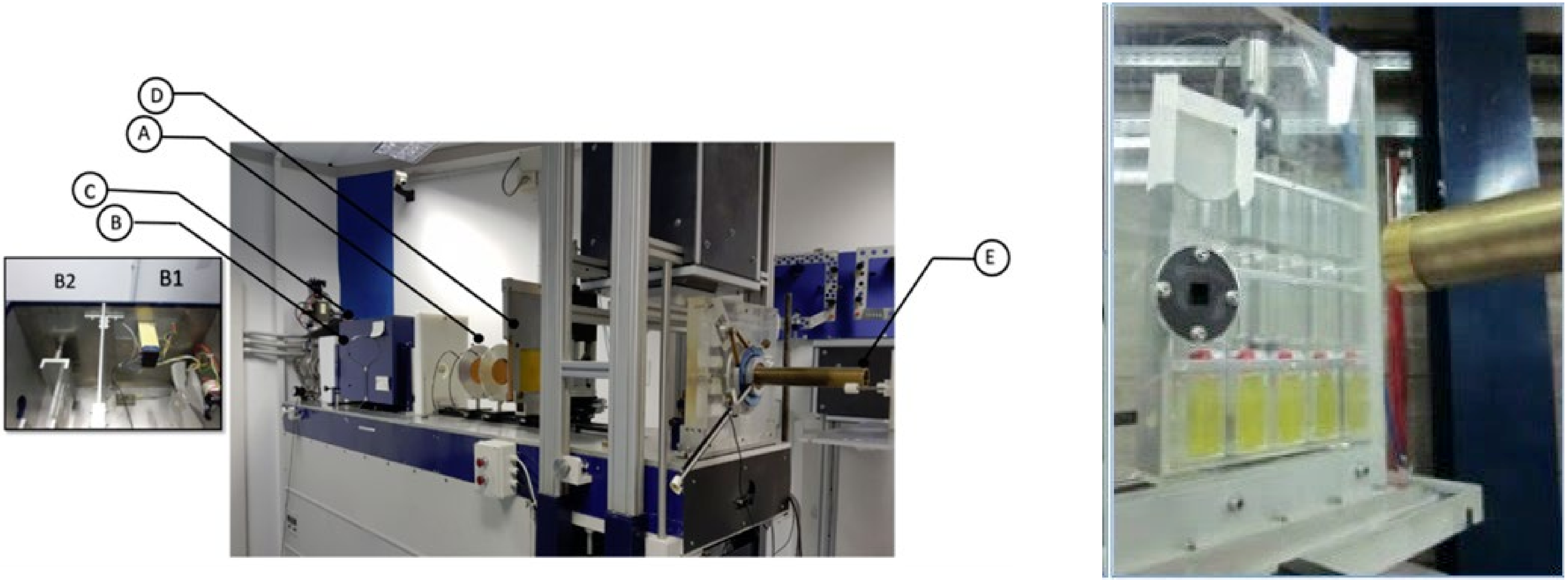

Supplementary Figure 1: Set-up for cellular irradiation at INFN-LNS. On the right, the CATANA proton beam line is shown, where **A.** Kapton window; **B.** Box encapsulating the energy modulator wheel **(B1)** and range shifter **(B2)**; **C.** Monitor chambers**; D.** The MOPI detector for online beam profile monitoring; **E.** End-of-line collimator. The two pictures (centre and left) show the mechatronic holder flask system, which allows to control remotely the movement of the sample holder in front of the beam collimator
