# Supplementary material for: The Proton-Boron Reaction Increases the Radiobiological Effectiveness of Clinical Low- and High-Energy Proton Beams: Novel Experimental Evidence and Perspectives
Source: Front Oncol. 2021 Jun 28;11:682647. doi: 10.3389/fonc.2021.682647 (PMC8274279; doi:10.3389/fonc.2021.682647)
Supplement: Supplementary file 2 [file DataSheet_2.pdf]

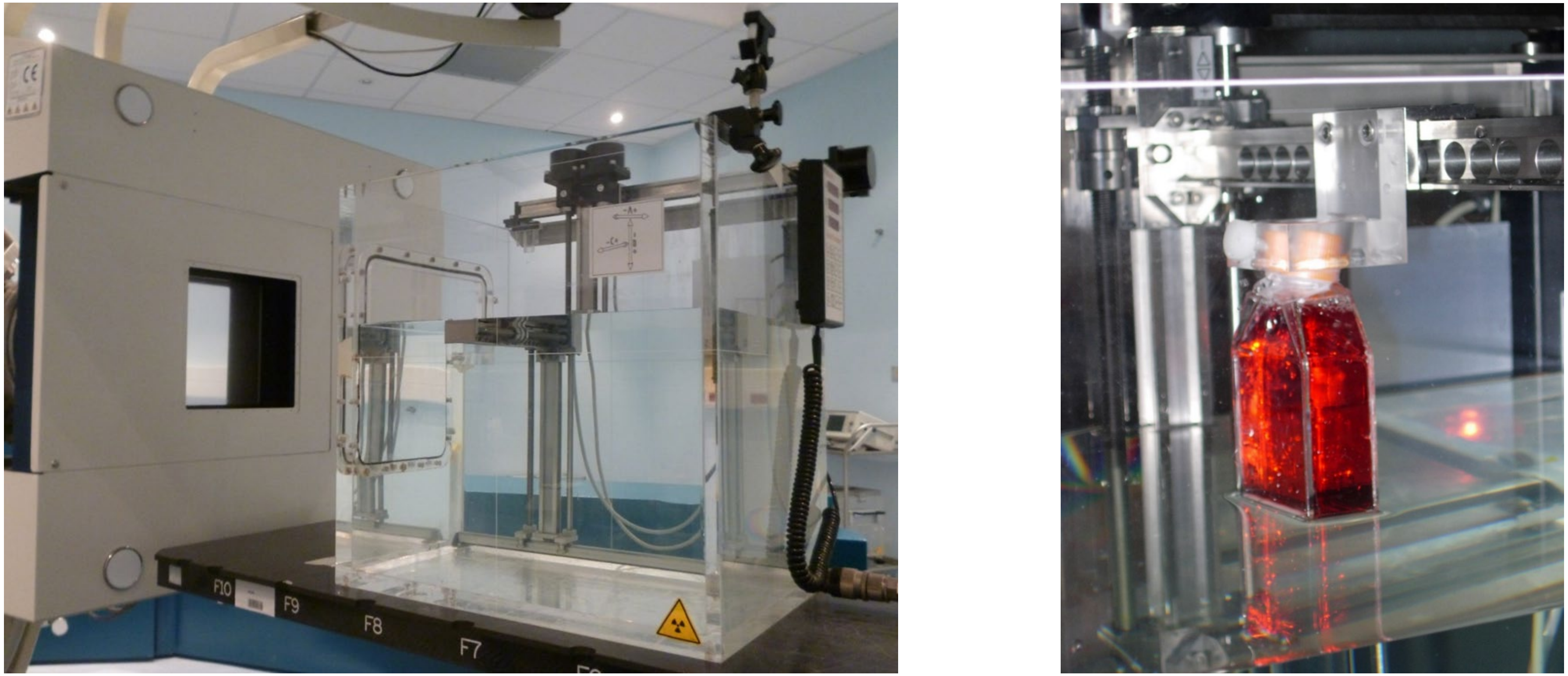

Supplementary Figure 2. Set-up for cellular irradiation used at the high-energy CNAO protontherapy beamline. On the left, next to the beam exit is shown the water tank where tissue culture flasks were placed for radiation exposure at different depths corresponding to various positions along the SOBP (see main text, Section see section Clinical High-Energy Proton Beamline). The picture on the right shows in detail a flask before being submerged held on to a motorized arm.
